# Molecular characterization of influenza A(H1N1)pdm09 in Cameroon during the 2014-2016 influenza seasons

**DOI:** 10.1371/journal.pone.0210119

**Published:** 2019-01-14

**Authors:** Chavely Gwladys Monamele, Hermann Landry Munshili Njifon, Marie-Astrid Vernet, Mohamadou Ripa Njankouo, Sebastien Kenmoe, Ali Ahmed Yahaya, Louis Deweerdt, Raphael Nono, Wilfred Mbacham, Damian Nota Anong, Jane Francis Akoachere, Richard Njouom

**Affiliations:** 1 National Influenza Centre, Centre Pasteur of Cameroon, Yaoundé, Cameroon; 2 University of Buea, Buea, Cameroon; 3 Centre Pasteur of Cameroon, Annex of Garoua, Garoua, Cameroon; 4 University of Yaoundé 1, Yaoundé, Cameroon; 5 World Health Organisation, Regional Office for Africa, Brazzaville, Congo; Erasmus University Medical Center, NETHERLANDS

## Abstract

In 2009, Influenza A(H1N1)pdm09 caused the first influenza pandemic of the 21st century with high mortality rates of about 284 500 deaths. This virus, however, continues to circulate as a seasonal influenza virus and to cause illness and deaths worldwide. In this study, we describe the genetic diversity of A(H1N1)pdm09 viruses collected between 2014 and 2016 in Cameroon. Three gene segments (HA, NA and M) of Cameroon strains were studied. The phylogenetic tree of the coding nucleotide sequences was generated by MEGA version 6.0 using a Maximum Likelihood method. The NA and M protein coding sequences were analyzed for the reported genetic markers of resistance against neuraminidase inhibitors and adamantanes, while predicted vaccine efficacy was estimated using the P_epitope_ method. Overall 39 strains were obtained. Phylogenetic analysis of the HA gene of influenza A(H1N1)pdm09 showed that Cameroon strains belonged to two major clades. The 2014 Cameroon sequences belonged to clade 6C while all sequences collected between 2015 and 2016 belonged to clade 6B. Majority of the samples had some mutations in the NA gene notably: I117M, N248D, and N369K while the amantadine-resistant M mutant, S31N, was found to be absent only in the two sequences collected in 2014. Overall, A/California/07/2009 vaccine strain showed a predicted vaccine efficacy of 24.55% to 35.77% against Cameroon A(H1N1)pdm09 strains circulating between 2014 and 2016. Our findings confirms the fast evolution of A(H1N1)pdm09 since its first introduction and highlights on the importance of influenza vaccine in reducing the burden caused by influenza in the community.

## Introduction

Influenza is an infectious viral disease and the major cause of acute respiratory disease in humans. Three worldwide outbreaks of influenza have occurred in the 20th century with high mortality rate of more than 40 million people [1, 2]. These outbreaks were respectively caused by the Spanish H1N1 in 1918, the Asian H2N2 in 1957, and the Hong Kong H3N2 in 1968 [1–3]. The 21st century has experienced an influenza pandemic in 2009 with 66 countries affected and over 284 500 deaths caused by the A(H1N1)pdm09 virus [4]. On August 2010, the end of the 2009 H1N1 influenza pandemic was declared by the World Health Organization (WHO). This virus however, continues to circulate as a seasonal influenza virus and to cause illness and deaths worldwide. Together with A(H3N2) and influenza B lineages, it accounts for approximately 290 000 to 650 000 deaths annually worldwide [5]

A(H1N1)pdm09 belongs to the *Orthomyxoviridae* family [6]. It contains eight negative-sense, single-stranded RNA segments named: PB2, PB1, PA, HA, NP, NA, M and NS1 in order of decreasing length [7]. The primary target of host neutralizing antibodies is the HA glycoprotein which mediates entry of the viral genome into the host cell [8, 9]. The HA molecules of influenza A(H1N1)pdm09 are known to have 4 distinct antigenic sites, Sa, Sb, Ca and Cb [10]. These sites consist of the most variable amino acids in the HA molecule that are subjected to antibody-mediated immune pressure. Continuous build-up of mutations at these antigenic sites is the basis for the evolutionary dynamics observed in the influenza virus resulting in antigenic drift [11]. Since the beginning of the 2009 pandemic, A(H1N1)pdm09 continuously evolved, and can be clustered into eight major genetic groups [12]. Thus, constant surveillance should be performed to monitor the evolution of circulating influenza virus.

From 2010 to 2016, WHO recommended A/California/07/2009 as the reference vaccine strain for A(H1N1)pdm09 virus and A/Michigan/45/2015 since 2017 [13]. The effectiveness of the influenza vaccine varies from year to year due to changes in the circulating influenza strains. Several methods have been proposed to estimate vaccine efficacy amongst which there is the P_epitope_ model which has been reported to be more efficacious than phylogenetic analyses or antisera hemagglutination inhibition assay [14]. P_epitope_ model quantifies the difference between dominant epitope regions in the vaccine and circulating strains and gives a measure which correlates well with influenza vaccine efficacy. The theoretical vaccine efficacy obtained with this method can be useful in circumstances where field investigation is not possible.

There has been previous reports on the genome characterization of influenza A(H1N1)pdm09 from respiratory samples collected between 2009 and 2013 [15]. In this study, we provide an update on the genome evolution of this relatively novel influenza virus between 2014 and 2016. Cameroon strains were either directly obtained from the National Influenza Centre at the Centre Pasteur of Cameroon (CPC) or downloaded from the Global Initiative on Sharing All Influenza Data (GISAID).

## Materials and methods

### Collection and preparation of samples

This study received approval from the Cameroon National Ethics committee N° 2016/08/798/CE/CNERSH/SP. Between January 2014 and June 2016, nasopharyngeal swabs were collected from patients with respiratory illness at twelve sites which are part of the influenza surveillance system in Cameroon. We included in this study, patients with influenza-like illness (ILI), or with severe acute respiratory infection (SARI) based on the WHO case definition as previously described [16]. Collected samples were stored at 4°C in a 2mL virus transport medium prior to analysis at the CPC. All samples were collected in accordance with relevant guidelines as written in the ethical clearance note (N° 2016/08/798/CE/CNERSH/SP).

### Nucleic acid extraction and detection of Influenza virus

Collected samples were screened for influenza virus following RNA extraction using the QIAamp Viral RNA Mini Kit (Qiagen, Hilden, Germany). Detection of influenza virus was performed using the real-time reverse transcription-polymerase chain reaction (RT-PCR) technology in an ABI prism 7300 or 7500 thermocycler (Applied Biosystems, Foster City, California, USA). The diagnostic kit used was the CDC Influenza A/B typing assay and the CDC H1/H3 sub-typing panel obtained through the International Reagent Resource Program (IRR, https://www.internationalreagentresource.org). Positive samples for influenza A were subsequently sub-typed for A(H1) and A(H3). Samples were considered positive for influenza A and A(H1N)2009 at a threshold cycle below 37 and only those with lower threshold cycles (below 30) were eligible for sequencing.

### Amplification of samples positive for A(H1N1)pdm09

Samples selected for further analysis were first amplified for the HA, NA and M gene with the Superscript III One Step RT-PCR System (Invitrogen, Carlsbad, California, USA) enzyme. Using a set of specific primers, 2 fragments of HA gene (1113bp and 967bp), 1 fragment each of NA gene (1418bp) and M gene (1027bp) were obtained. S1 Table gives details of the nucleotide composition of the primers used. A 50 μL reaction mixture was composed of 25 μL of 2x PCR buffer, 0.4 μM forward and reverse primers, 0.19 U/μL RNAsin, 2μL enzyme and 10μL RNA. The amplification of the 1113bp fragment of the HA gene used the program: 45°C for 30min, 55°C for 15min, 94°C for 2min, 35 cycles at (94°C for 45sec, 52.5°C for 45sec, 72°C for 75sec) and 72°C for 5min. The following thermal cycling conditions was used for amplification of the 967bp fragment of the HA gene: 45°C for 30min, 55°C for 15min, 94°C for 2min, 37 cycles at (94°C for 45sec, 48°C for 45sec, 72°C for 75sec) and 72°C for 5min. For the 1418bp fragment of the NA gene, the following program was used: 45°C for 30min, 55°C for 15min, 94°C for 2min, 33 cycles at (94°C for 45sec, 60°C for 45sec, 72°C for 90sec) and 72°C for 5min. While for the M gene, the program used was: 45°C for 30min, 55°C for 15min, 94°C for 2min, 42 cycles at (94°C for 45sec, 45°C for 45sec, 72°C for 75sec) and 72°C for 5min. Amplicons of the appropriate size after gel electrophoresis were sent for sequencing at GENEWIZ UK, LTD (Hope End, UK).

### Genome characterization of A(H1N1)pdm09

Raw sequencing data obtained from GENEWIZ UK, LTD (Hope End, UK) were edited and assembled using CLC Main Workbench version 5.5. Cameroon strains sequences were aligned with other relevant strains sequences obtained from online databases (GenBank and GISAID), notably A/California/07/2009 [13], the WHO recommended vaccine strain for the 2010–2016 Influenza seasons. Evolutionary trees were generated in MEGA version 6.0 using the Maximum Likelihood method and the Kimura 2-parameter model for estimating distances. We used 1000 bootstrap replicates in generating the phylogenetic trees and labelled branches with high bootstrap values above 70%. Amino acid identity was calculated with A/California/07/2009 as reference. Analysis of the amino acid identity for the three gene segments was performed using flusurver (http://flusurver.bii.a-star.edu.sg) with A/California/07/2009 as reference. Mutations in the three gene segments were reported with respect to the H3 numbering system (without the signal peptide).

### Prediction of potential glycosylation sites

Potential N-linked glycosylation sites were predicted using the NetNGlyc 1.0. online server (http://www.cbs.dtu.dk/services/NetNGlyc/) [17]. This server predicts glycosylation sites for amino acid alignments Asparagine-X-Serine/Threonine, where X can be any amino acid except Aspartic acid or Proline. Threshold values above 0.5 suggest glycosylation.

### Estimating vaccine efficacy of A(H1N1)pdm09 strains using P_epitope_ model

The predicted vaccine efficacy of A(H1N1)pdm09 was estimated using P_epitope_ which is a measure of the fractional change in the amino acid substitutions between the dominant epitope of the vaccine strain and the circulating strain [14]. In order to determine antigenic drift and measure predicted vaccine efficacy, we considered five epitope regions (A-E) in the (H1N1)pdm09 strains in analogy to the A(H3N2) virus as previously reported [18]. The following formula was used in calculating P_epitope_: number of mutations in dominant epitope/number of amino acids in dominant epitope [14]. The dominant epitope was considered to be the epitope with the highest percentage of mutations. Predicted vaccine efficacy (E) was measured from P_epitope_ with the equation, E = (0.47–2.47 x P_epitope_) x 100 [18].

## Results

### Characteristics of A(H1N1)pdm09 strains

There were 23 samples randomly selected from CPC for HA, NA and M gene sequencing and clean sequences were obtained from 17 samples (17 HA, 16 NA, 14 M genes). Additionally, all Cameroon strains collected during the same period of January 2014 to June 2016 were retrieved from the GISAID database. Overall 39 sequences were obtained from both sources. Two were collected in 2014, 30 in 2015 and 7 in 2016. S2 Table gives detailed characteristics of influenza A(H1N1)pdm09 strains from Cameroon. Influenza A(H1N1)pdm09 sequences obtained in this study were submitted to Genbank under accession numbers MH168280-MH168323 and MH920242-MH920245.

### Surface glycoprotein genes and gene segments

#### Haemagglutinin

**Fig 1:**
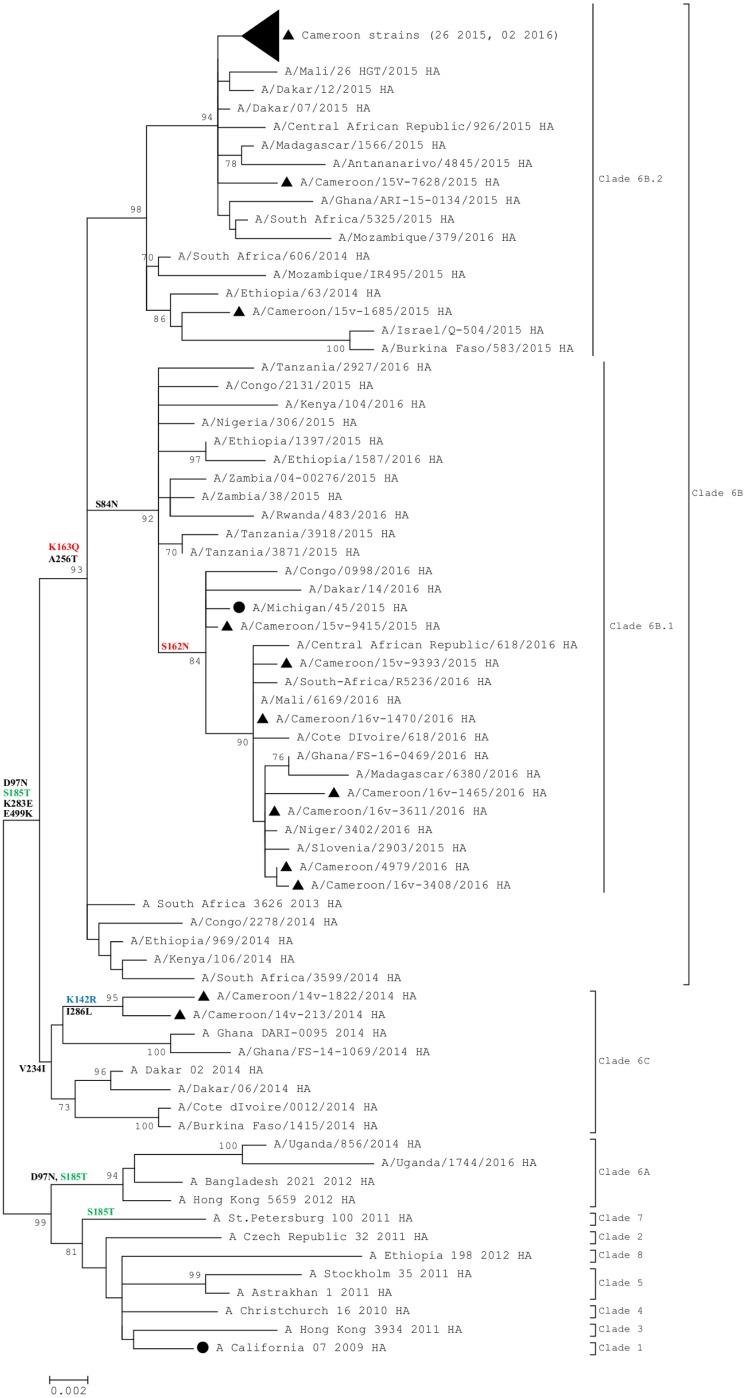
Phylogenetic analysis of HA nucleotide sequences of Influenza A(H1N1)pdm09. **●** represents the northern hemisphere vaccine strains recommended by WHO and ▲ represents Cameroon strains. In colour are substitutions in antigenic sites: Ca (blue), Sa (red) and Sb (green). The evolutionary history was inferred by using the Maximum Likelihood method based on the Kimura 2-parameter model. The percentage of trees in which the associated taxa clustered together is shown next to the branches.

In order to determine the clade distribution of Cameroon sequences, we performed phylogenetic analysis of the HA gene of influenza A(H1N1)pdm09 viruses. Analysis revealed that the sequences belonged to two major clades. The two sequences collected in 2014 belonged to clade 6C represented by A/Dakar/02/2014 while all sequences collected between 2015 and 2016 belonged to clade 6B represented by A/South Africa/3626/2013 (Fig 1). Cameroon sequences of clade 6B further grouped into two distinct clusters represented by A/Slovenia/2903/2015 and A/Israel/Q-504/2015.

Cameroon strains (2014–2016) shared 98.5 to 99.1% nucleotide sequence identity and 97.2–98.7% amino acid sequence identity with the A/California/07/2009 vaccine strain. The 2014 virus strains differed from the vaccine strain, by possessing 3 amino acid substitutions: K142R (antigenic site Ca), V234I and I286L. Whereas, 2015 and 2016 Cameroon strains differed from the vaccine strain by possessing the substitutions K163Q (antigenic site Sa) and A256T. Amino acid residues in antigenic sites Sa, Sb, Ca, and Cb of the globular head are defined according to a previous report [10].

#### Neuraminidase

**Fig 2:**
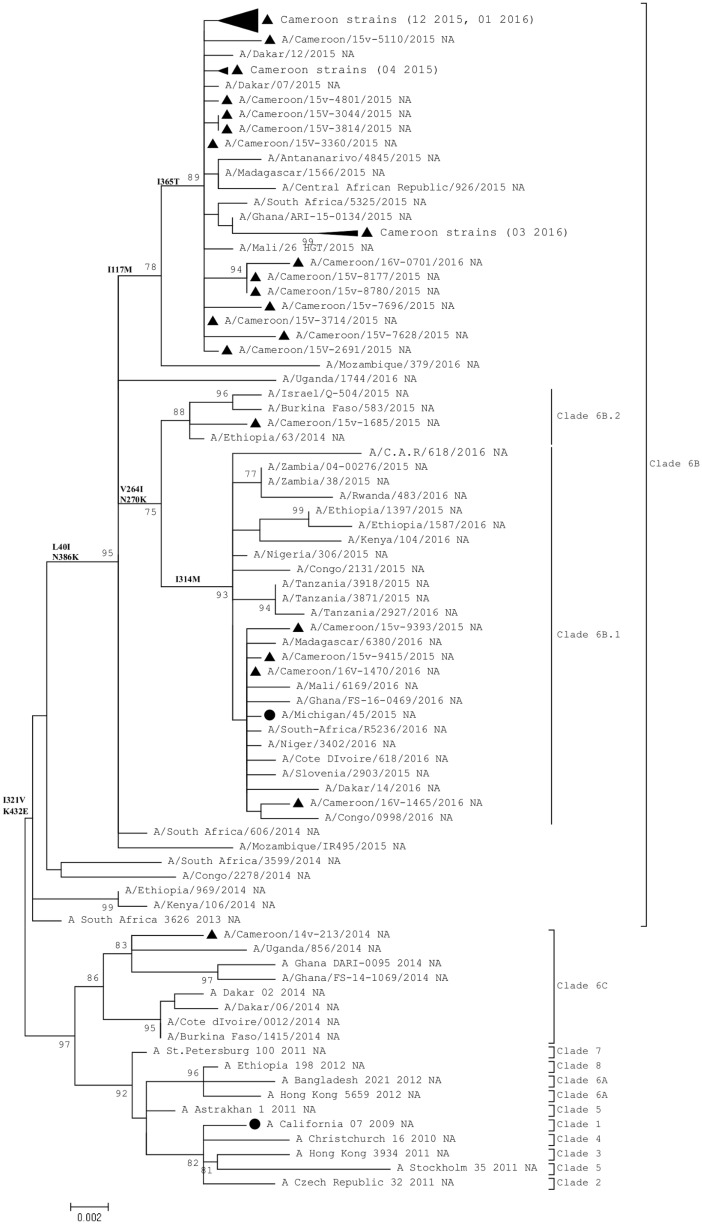
Phylogenetic analysis of NA nucleotide sequences of Influenza A(H1N1)pdm09. ● represents the Northern hemisphere vaccine strains recommended by WHO and ▲ represents Cameroon strains. The evolutionary history was inferred by using the Maximum Likelihood method based on the Kimura 2-parameter model. The percentage of trees in which the associated taxa clustered together is shown next to the branches.

The NA sequence analysis showed that all Cameroon strains from the 2015–2016 seasons had evolved away from the A/South Africa/3626/2013 virus (Fig 2) while the strains from 2014 influenza season clustered with the A/Dakar/02/2014.

The NA genes of the 2015/2016 virus strains differed from those of the 2014 virus strains by substitutions of several amino acids.

### Antiviral drug susceptibility profile of Cameroon strains

The NA and M2 coding sequences of the A(H1N1)pdm09 strains were analyzed for resistance-inducing mutations against the two known classes of drugs: neuraminidase inhibitors (NAIs) and adamantanes. None of the strains possessed the H275Y mutation that has been reported clinically to cause highly reduced inhibition to NAIs [19, 20]. None of the Cameroon strains possessed mutations that are associated with reduced inhibition in vitro by NAIs, particularly at positions 117, 119, 136, 151, 152, 199, 223, 247, 293 and 295 [20]. We however noted some mutations in the NA gene following the results from FluSurver analyses, notably: I117M (84.2%), N248D (94.7%), and N369K (94.7%).

Viruses with resistance to adamantanes generally have mutations located at positions 26, 27, 30 and 31 of the M2 protein [21]. The amino acid substitution S31N is the predominant amantadine-resistant M2 mutant and was found to be present in almost all of the circulating influenza A(H1N1)pdm09 strains except the two strains collected in 2014.

### Measurement of predicted vaccine efficacy

Amino acid residues in the five epitope regions A to E are defined according to a previous report [18] to respectively possess 24, 22, 33, 48, and 34 amino acids. Table 1 gives an estimate of the predicted vaccine efficacy of A/California/07/2009 vaccine strain in Cameroon during the 2014 to 2016 influenza seasons. For 2014, the P_epitope_ between A(H1N1)pdm09 strains and the A/California/07/2009 vaccine strain was 0.059 (epitope E), which suggests a predicted vaccine efficacy against these strains of 32.47% of that of a perfect match with the vaccine strain. For the 2015 influenza season, antigenic drift was mainly observed on epitope A while for the 2016 influenza season, mutations were mostly observed on epitope B, with an estimated predicted vaccine efficacy ranging between 24.55% and 35.77% for both seasons.

**Table 1:** Predicted vaccine efficacy of A/California/07/2009 vaccine strain during the 2014 to 2016 influenza seasons in Cameroon.

Year (N)	No. of strains	Epitope	No. of mutations	Residue differences	P_epitope_	Vaccine efficacy (%)
2014 (N = 2)	2	A	1	K142R	0.042	36.71
	2	B	1	S185T	0.045	35.77
	2	C	1	K283E	0.030	39.52
	0	D	0	n/a	n/a	n/a
	**2**	E*	**2**	**A48S, P83S**	**0.059**	**32.47**
2015 (N = 30)	**30**	A*	**2**	**T120A, A141V**	**0.083**	**26.42**
	30	B	1	S185T	0.045	35.77
	**8**	B*	**2**	**S162R/N, S185T**	**0.091**	**24.55**
	30	C	1	K283E	0.030	39.52
	3	C	2	H273N, K283E	0.061	32.03
	30	D	1	K163Q	0.021	41.85
	2	D	2	K163Q, I216T	0.042	36.71
	28	E	1	P83S	0.029	39.74
	2	E	2	P83S, S84N	0.059	32.47
2016 (N = 7)	0	A	0	n/a	n/a	n/a
	**7**	B*	**1**	**S185T**	**0.045**	**35.77**
	**5**	B*	**2**	**S162N, S185T**	**0.091**	**24.55**
	7	C	1	K283E	0.030	39.52
	7	D	1	K163Q	0.021	41.85
	4	D	2	K163Q, I216T	0.042	36.71
	7	E	1	P83S	0.029	39.74
	5	E	2	P83S, S84N	0.059	32.47

Predicted vaccine efficacy (E) was calculated using the formula E = (0.47–2.47 x P_epitope_) x 100. Dominant epitopes are designated by (*). n/a = not applicable.

### Prediction of glycosylation sites

There were 6 predicted glycosylation sites in the HA gene of all strains: 11(NST), 23(NVT), 87(NGT), 287(NTS), 481(NGT) and 540(NGS). Some strains had additional glycosylation sites with lower threshold values (0.5–0.7) at positions 10 (NNS; 2/39), 162 (NQS; 7/39) and 276 (NTT; 1/39). Similarly, A/California/07/2009 vaccine strain possessed the 6 glycosylation sites common to all Cameroon strains.

## Discussion

Analysis of the gene segments of influenza A(H1N1)pdm09 viruses from Cameroon showed similar phylogenetic clusters in the HA and NA genes. Two major clades were found to circulate in Cameroon between 2014 and 2016. The two sequences collected in 2014 belonged to clade 6C represented by A/Dakar/02/2014 while all sequences collected between 2015 and 2016 belonged to clade 6B represented by A/South Africa/3626/2013. Several other studies have shown the same temporal circulation of these two clades [22, 23] while others showed the presence of viruses of clade 6B in the 2014 season [24–26]. The 2014 virus strains differed from the vaccine strain by acquiring an amino acid substitution on antigenic site Ca (K142R). Whereas, 2015 and 2016 Cameroon strains possessed mutations on antigenic sites Sa (S162N, K163Q) and Sb (S185T). A previous study showed that A(H1N1)pdm09 strains from Cameroon including several African countries between 2009 and 2013 fell into a distinct clade denoted by West Africa clade II in circulation for almost two years [15]. The fact that the studied viruses formed two distinct clusters with different epitope dominance highlights the ongoing evolution of this virus despite its relatively delayed apparition in Africa (6 months after the emergence of the virus) and probably late detection.

Cameroon strains were found to be susceptible to NAIs based on genotypic drug resistance testing. These results are however different from that noted on H1N1 strains collected between 2007 and 2008 from Cameroon in which the H275Y mutation peculiar of resistance to NAIs was observed despite the non-exposition to this antiviral [27]. This can be explained by the fact that A(H1N1)pdm09 is a relatively novel strain and has not been subjected to much antiviral pressure. However, some substitutions in the NA gene (I117M, N248D, and N369K) as recorded with the FluSurver (http://flusurver.bii.a-star.edu.sg) might be involved in drug susceptibility. Experimental testing is required to confirm the presence of these resistance-causing mutations due to its public health implication in the control of A(H1N1)pdm09 viruses. Meanwhile, the amino acid substitution S31N in the M2 protein was found to be present in all but the 2014 strains. These results are similar to several other studies where high prevalence of amantadine resistance was observed in influenza A viruses irrespective of its use [28].

Estimates of vaccine efficacy of A/California/07/2009 in Cameroon from 2014–2016 showed a predicted vaccine efficacy between 24.55% and 35.77% against circulating A(H1N1)pdm09 strains. This estimate is similar to that observed from other studies. In the United States, where vaccine coverage for influenza virus is good, the vaccine effectiveness estimates for the 2014–2016 influenza seasons ranged 19% to 48% based on an observational study design [29]. Similar estimates of below 50% were obtained in Thailand with this same method though it was during the 2010–2014 influenza seasons [24]. The P_epitope_ method has proven to be an alternative method for estimating vaccine efficacy. Moreover, it shows how close the circulating strains are to the vaccine strains and emphasize on the importance of considering genetic data from around the world for the selection of the flu vaccine.

Overall, the predicted vaccine efficacy for influenza A(H1N1)pdm09 was higher than that for A(H3N2) within the same influenza season in Cameroon with P_epitope_ ranging 0.05–0.21 for influenza A(H3N2) versus 0.045–0.091 for A(H1N1)pdm09 [16]. Previous studies have shown that vaccine efficacy for A(H1N1)pdm09 is generally higher than that for A(H3N2) [24, 30]. This phenomenon could be explained by the fact that there is higher susceptibility to the relatively novel A(H1N1)pdm09 strain, leading to weaker immune pressure and lower rates of viral evolution as compared to A(H3N2) virus [24]. These results highlight the importance of sensitizing the population on the uptake of influenza vaccine in order to reduce the burden caused by this infection in Cameroon.

Similarly to the vaccine strain, A/California/07/2009, there were 4 predicted glycosylation sites in the HA gene of all Cameroon A(H1N1)pdm09 viruses. Some Cameroon strains had two additional glycosylation sites, 10(NNS) and 162(NQS), not found in the vaccine strain: The glycosylation site 162(NQS) is the consequence of the amino acid substitution S162N found in viruses of sub-clade 6B.1 represented mainly by the 2016 viruses. A reduced predicted vaccine efficacy of 24.55% was observed in viruses belonging to this sub-clade. As reported by Mostafa et al., glycosylation and deglycosylation patterns are viral mechanisms that can mask antigenic epitopes and thus inhibit binding to neutralizing antibodies [31]. This is a possible explanation to the diminished predicted vaccine efficacy noted in viruses which acquired the 162(NQS) glycosylation site.

A main limitation to this study was the non-representativity of the study strains with regards to circulating strains especially in 2014 where sequences were obtained from only two samples. Indeed, genome characterization was realized in approximately 32% (39/122) of the overall positive samples identified while CPC sequenced strains accounted for about 43% of the studied viruses. Another limit to this study was the fact that there is no consensus method for P_epitope_ calculation in influenza A(H1N1)pdm09. We calculated Pepitope based on a previous definition, and estimated predicted vaccine efficacy with respect to observed mutations on the dominant epitope.

## Conclusion

Our findings confirms the fast evolution of influenza A(H1N1)pdm09 virus since its first introduction and the importance of continuously performing molecular surveillance of circulating influenza virus. This study also highlights on the importance of influenza vaccine in reducing the burden caused by influenza in the community.

## Supporting information

S1 Table Set of primers for PCR and sequencing. (DOCX)

S2 Table Characteristics of influenza A(H1N1)pdm09 strains obtained in this study. (DOCX)
