# Supplementary material for: Molecular characterization of influenza A(H1N1)pdm09 in Cameroon during the 2014-2016 influenza seasons
Source: PLoS One. 2019 Jan 14;14(1):e0210119. doi: 10.1371/journal.pone.0210119 (PMC6331099; doi:10.1371/journal.pone.0210119)
Supplement: S1 Table — (DOCX) [file pone.0210119.s001.docx]

| Gene | Oligo name | Primer sequences 5’-3’ | Expected size (bp) |
| --- | --- | --- | --- |
| Hemagglutinin (H1) | H1p/4F | AAAAGCAGGGGAAAACAAAAG | 1113 |
|  | H1p/1117R | ATCATTCCAGTCCATCCCCTTCAAT |  |
|  | H1p/803F | AACATTCGAAGCAACTGGAAAT | 967 |
|  | H1p/1770R | ACAAGGGTGTTTTTCTCATGCT |  |
| Neuraminidase (N1) | N1p/7F | AGCAGGAGTTTAAAATGAATCC | 1418 |
|  | N1p/720R | CACAGACACATTCAGACTCTTG |  |
|  | N1p/1425R | TGTCAATGGTAAATGGCAACTC |  |
| Matrix (M) | M/1F | AGCAAAAGCAGGTAGATATTGA | 1027 |
|  | M/237F | AGCGAGGACTGCAGCGTAG |  |
|  | M/841R | GAATCCACAATATCAAGTGCA |  |
|  | M/1027R | AGTAGAAACAAGGTAGTTTTTTACTC |  |
