# Supplementary material for: Molecular characterization of influenza A(H1N1)pdm09 in Cameroon during the 2014-2016 influenza seasons
Source: PLoS One. 2019 Jan 14;14(1):e0210119. doi: 10.1371/journal.pone.0210119 (PMC6331099; doi:10.1371/journal.pone.0210119)
Supplement: S2 Table — (DOCX) [file pone.0210119.s002.docx]

| **Sequence Name** | **Collection month** | **Age (year)** | **Sex** | **Gene** | **Accession number** |
| --- | --- | --- | --- | --- | --- |
| **CPC sequences** | | | | | |
| A/Cameroon/14v-213/2014 | January | 26 | M | HA | MH168281 |
| A/Cameroon/14v-213/2014 | January | 26 | M | NA | MH168301 |
| A/Cameroon/14v-213/2014 | January | 26 | M | M | MH168316 |
| A/Cameroon/14v-1822/2014 | March | 23 | F | HA | MH168280 |
| A/Cameroon/14v-1822/2014 | March | 23 | F | NA | MH168300 |
| A/Cameroon/14v-1822/2014 | March | 23 | F | M | MH168315 |
| A/Cameroon/15v-1685/2015 | February | 29 | F | HA | MH168282 |
| A/Cameroon/15v-1685/2015 | February | 29 | F | NA | MH168302 |
| A/Cameroon/15v-4801/2015 | April | 4 | F | HA | MH920243 |
| A/Cameroon/15v-4801/2015 | April | 4 | F | NA | MH920245 |
| A/Cameroon/15v-5110/2015 | June | 0 (9 months) | F | HA | MH168283 |
| A/Cameroon/15v-5110/2015 | June | 0 (9 months) | F | NA | MH168303 |
| A/Cameroon/15v-5110/2015 | June | 0 (9 months) | F | M | MH168317 |
| A/Cameroon/15v-5112/2015 | June | 2 | M | HA | MH168284 |
| A/Cameroon/15v-5112/2015 | June | 2 | M | NA | MH168304 |
| A/Cameroon/15v-5112/2015 | June | 2 | M | M | MH168318 |
| A/Cameroon/15v-5447/2015 | July | 0 (7 months) | F | HA | MH168285 |
| A/Cameroon/15v-5447/2015 | July | 0 (7 months) | F | NA | MH168305 |
| A/Cameroon/15v-5447/2015 | July | 0 (7 months) | F | M | MH168319 |
| A/Cameroon/15v-5879/2015 | August | 1 | F | HA | MH168286 |
| A/Cameroon/15v-5879/2015 | August | 1 | F | NA | MH168306 |
| A/Cameroon/15v-5879/2015 | August | 1 | F | M | MH168320 |
| A/Cameroon/15v-6593/2015 | September | 3 | M | HA | MH168287 |
| A/Cameroon/15v-6593/2015 | September | 3 | M | NA | MH168307 |
| A/Cameroon/15v-6593/2015 | September | 3 | M | M | MH168321 |
| A/Cameroon/15v-7696/2015 | September | - | - | HA | MH920242 |
| A/Cameroon/15v-7696/2015 | September | - | - | NA | MH920244 |
| A/Cameroon/15v-8780/2015 | November | 1 | M | HA | MH168288 |
| A/Cameroon/15v-8780/2015 | November | 1 | M | NA | MH168308 |
| A/Cameroon/15v-8780/2015 | November | 1 | M | M | MH168322 |
| A/Cameroon/16v-027/2016 | December | 4 | F | HA | MH168289 |
| A/Cameroon/16v-027/2016 | December | 4 | F | NA | MH168309 |
| A/Cameroon/16v-027/2016 | December | 4 | F | M | MH168323 |
| A/Cameroon/16v-0701/2016 | February | 23 | M | HA | MH168290 |
| A/Cameroon/16v-0701/2016 | February | 23 | M | NA | MH168295 |
| A/Cameroon/16v-0701/2016 | February | 23 | M | M | MH168310 |
| A/Cameroon/16v-1465/2016 | March | 24 | F | HA | MH168291 |
| A/Cameroon/16v-1465/2016 | March | 24 | F | NA | MH168296 |
| A/Cameroon/16v-1465/2016 | March | 24 | F | M | MH168311 |
| A/Cameroon/16v-1470/2016 | March | 69 | M | HA | MH168292 |
| A/Cameroon/16v-1470/2016 | March | 69 | M | NA | MH168297 |
| A/Cameroon/16v-1470/2016 | March | 69 | M | M | MH168312 |
| A/Cameroon/16v-3408/2016 | April | 29 | F | HA | MH168293 |
| A/Cameroon/16v-3408/2016 | April | 29 | F | NA | MH168298 |
| A/Cameroon/16v-3408/2016 | April | 29 | F | M | MH168313 |
| A/Cameroon/16v-3611/2016 | May | 1 | M | HA | MH168294 |
| A/Cameroon/16v-3611/2016 | May | 1 | M | NA | MH168299 |
| A/Cameroon/16v-3611/2016 | May | 1 | M | M | MH168314 |
| **GISAID sequences** | | | | | |
| A/Cameroon/15v-2449/2015 | March | 4 | M | HA | EPI630604 |
| A/Cameroon/15v-2449/2015 | March | 4 | M | NA | EPI630605 |
| A/Cameroon/15v-2682/2015 | March | 43 | M | HA | EPI630606 |
| A/Cameroon/15v-2682/2015 | March | 43 | M | NA | EPI630607 |
| A/Cameroon/15v-2691/2015 | March | 0 (4 months) | M | HA | EPI630608 |
| A/Cameroon/15v-2691/2015 | March | 0 (4 months) | M | NA | EPI630609 |
| A/Cameroon/15v-2860/2015 | April | 21 | F | HA | EPI624667 |
| A/Cameroon/15v-2860/2015 | April | 21 | F | NA | EPI624668 |
| A/Cameroon/15v-3038/2015 | April | 2 | M | HA | EPI630610 |
| A/Cameroon/15v-3038/2015 | April | 2 | M | NA | EPI630611 |
| A/Cameroon/15v-3044/2015 | April | 2 | M | HA | EPI630612 |
| A/Cameroon/15v-3044/2015 | April | 2 | M | NA | EPI630613 |
| A/Cameroon/15v-3360/2015 | April | - | - | HA | EPI630614 |
| A/Cameroon/15v-3360/2015 | April | - | - | NA | EPI630615 |
| A/Cameroon/15v-3714/2015 | May | 1 | M | HA | EPI624669 |
| A/Cameroon/15v-3714/2015 | May | 1 | M | NA | EPI624670 |
| A/Cameroon/15v-3719/2015 | May | 7 | F | HA | EPI624671 |
| A/Cameroon/15v-3719/2015 | May | 7 | F | NA | EPI624672 |
| A/Cameroon/15v-3814/2015 | May | 3 | F | HA | EPI624673 |
| A/Cameroon/15v-3814/2015 | May | 3 | F | NA | EPI629043 |
| A/Cameroon/15v-4100/2015 | May | 1 | M | HA | EPI624674 |
| A/Cameroon/15v-4100/2015 | May | 1 | M | NA | EPI624675 |
| A/Cameroon/15v-5175/2015 | July | 2 | F | HA | EPI748826 |
| A/Cameroon/15v-5175/2015 | July | 2 | F | NA | EPI748827 |
| A/Cameroon/15v-7628/2015 | October | 43 | F | HA | EPI748828 |
| A/Cameroon/15v-7628/2015 | October | 43 | F | NA | EPI748829 |
| A/Cameroon/15v-8162/2015 | November | 3 | M | HA | EPI748830 |
| A/Cameroon/15v-8162/2015 | November | 3 | M | NA | EPI748831 |
| A/Cameroon/15v-8177/2015 | November | 3 | M | HA | EPI748832 |
| A/Cameroon/15v-8177/2015 | November | 3 | M | NA | EPI748833 |
| A/Cameroon/15v-8406/2015 | November | 2 | M | HA | EPI748834 |
| A/Cameroon/15v-8406/2015 | November | 2 | M | NA | EPI748835 |
| A/Cameroon/15v-8802/2015 | November | 1 | F | HA | EPI748836 |
| A/Cameroon/15v-8802/2015 | November | 1 | F | NA | EPI748837 |
| A/Cameroon/15v-9393/2015 | December | 0 (1 month) | M | HA | EPI706886 |
| A/Cameroon/15v-9393/2015 | December | 0 (1 month) | M | NA | EPI706887 |
| A/Cameroon/15v-9415/2015 | December | 40 | F | HA | EPI706888 |
| A/Cameroon/15v-9415/2015 | December | 40 | F | NA | EPI706889 |
| A/Cameroon/GARO 67/2015 | September | - | - | HA | EPI748838 |
| A/Cameroon/GARO 67/2015 | September | - | - | NA | EPI748839 |
| A/Cameroon/NGHP 68/2015 | August | - | - | HA | EPI748840 |
| A/Cameroon/NGHP 68/2015 | August | - | - | NA | EPI748841 |
| A/Cameroon/4979/2016 | June | - | - | HA | EPI858903 |
| A/Cameroon/4979/2016 | June | - | - | NA | EPI858904 |
